# Circulating calcification inhibitors are associated with arterial damage in pediatric patients with primary hypertension

**DOI:** 10.1007/s00467-021-04957-5

**Published:** 2021-02-18

**Authors:** Piotr Skrzypczyk, Anna Stelmaszczyk-Emmel, Michał Szyszka, Anna Ofiara, Małgorzata Pańczyk-Tomaszewska

**Affiliations:** 1 Department of Pediatrics and Nephrology, Medical University of Warsaw, ul. Żwirki i Wigury 63A, 02-091 Warsaw, Poland; 2 Department of Laboratory Diagnostics and Clinical Immunology of Developmental Age, Medical University of Warsaw, ul. Żwirki i Wigury 63A, 02-091 Warsaw, Poland

**Keywords:** Primary hypertension, Children, Arterial damage, Calcification inhibitors, Fetuin A

## Abstract

**Background:**

Circulating calcification inhibitors: fetuin A (FA) and osteoprotegerin (OPG) together with soluble ligand of receptor activator of nuclear factor kappa-B (sRANKL) have been linked to vascular calcifications and arterial damage. This study aimed to evaluate relationships between FA, OPG, sRANKL, and arterial damage in children with primary hypertension (PH).

**Methods:**

In this cross-sectional single-center study, calcification inhibitors (FA, OPG, sRANKL) levels were measured in blood samples of 60 children with PH (median age 15.8, IQR: [14.5–16.8] years) and 20 age-matched healthy volunteers. In each participant, peripheral and central blood pressure evaluation (BP) and ambulatory BP monitoring (ABPM) were performed. Arterial damage was measured using common carotid artery intima media thickness (cIMT), pulse wave velocity (PWV), augmentation index (AIx75HR), and local arterial stiffness (ECHO-tracking—ET) analysis.

**Results:**

Children with PH had significantly higher peripheral and central BP, BP in ABPM, thicker cIMT, higher PWV, and AIx75HR. FA was significantly lower in patients with PH compared to healthy peers without differences in OPG, sRANKL, and OPG/sRANKL and OPG/FA ratios. In children with PH, FA level correlated negatively with cIMT Z-score and ET AIx; sRANKL level correlated negatively with ABPM systolic blood pressure (SBP), SBP load, diastolic BP load, and AIx75HR; OPG/sRANKL ratio correlated positively with SBP load, while OPG/FA ratio correlated positively with ET AIx. In multivariate analysis, FA was a significant determinant of cIMT (mm) and cIMT Z-score.

**Conclusions:**

This study reveals that in children with primary hypertension, arterial damage is related to lower fetuin A concentrations.

**Graphical abstract:**

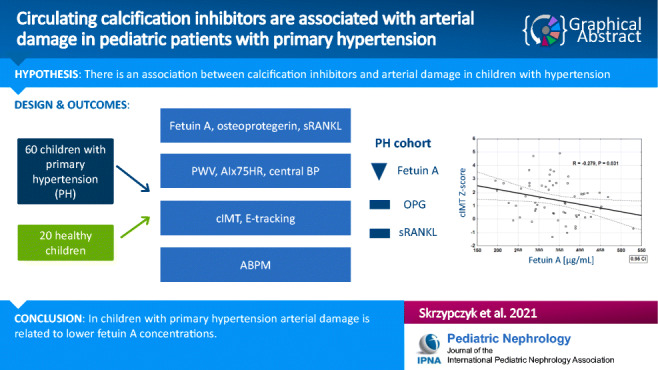

**Supplementary Information:**

The online version contains supplementary material available at 10.1007/s00467-021-04957-5.

## Introduction

Primary hypertension (PH) is a complex systemic disorder involving in its pathogenesis genetic factors, environmental factors (e.g., sedentary lifestyle, high-sodium diet), inappropriate body composition and fatty tissue distribution, dysregulation of sympathetic, immune, and renin-angiotensin-aldosterone systems, endothelial dysfunction, gut microbiota dysbiosis, as well as premature (early) vascular aging [1].

Increased arterial stiffness and abnormal common carotid artery intima-media thickness (cIMT) have been recognized as independent risk factors for cardiovascular morbidity and mortality in adult populations [2, 3]. However, increased arterial stiffness and cIMT abnormalities are not uncommon in pediatric patients with PH [4]. Recent experimental studies indicate that arterial lesions are associated with endothelial dysfunction, subclinical inflammation, oxidative stress, altered smooth muscle cell number, structure and function, and vascular calcification processes [5]. Vascular calcification is an active, cell-regulated process, which may substantially worsen arterial damage, with numerous molecules involved.

Fetuin-A (FA) (α2-Heremans-Schmid glycoprotein) is a soluble glycoprotein produced exclusively by hepatocytes and released into the circulation in high concentrations. It is involved in several functions, such as endocytosis, brain development, insulin resistance, and formation of bone tissue, and shows negative correlations with systemic and local inflammation. FA, considered the most potent systemic inhibitor of calcification, forms a soluble complex comprised of 8 FA molecules, 790 calcium atoms, and 58 phosphate molecules (calciprotein), protecting against formation of extraosseous calcium deposits [6, 7].

Osteoprotegerin (OPG) is a soluble glycoprotein, found as either a 60 kDa monomer or 120 kDa dimer, produced by osteoblasts, epithelial cells, vascular endothelial cells, as well as B cells and dendritic cells. In bone tissue, OPG acts as a decoy receptor for soluble ligand of the receptor activator of nuclear factor kappa-B (sRANKL). Once released by osteoblasts, sRANKL binds to RANK receptor on the surface of osteoclast progenitor cells, thus activating the nuclear factor kappa B (NF-κB) and triggering osteoclast precursor cell differentiation into mature osteoclasts, which in turn increases bone resorption. Recently, OPG was found to exert numerous systemic, extraosseous actions including inhibition of soft tissue and vascular calcifications [8, 9].

Studies in vitro and in animal models suggest that both OPG and FA inhibit vascular calcification [10, 11]. Numerous adult data on the significance of these molecules as biomarkers of subclinical arterial damage in patients with chronic kidney disease (CKD) [12], coronary artery disease [13], and PH [14, 15] give inconsistent results. There are only a few pediatric studies analyzing relationships between cIMT or arterial stiffness and the aforementioned biomarkers [16–20].

To the best of our knowledge, there is no data in the literature analyzing dependence between target-organ damage, FA, OPG, and sRANKL in pediatric patients with PH. Thus, the aim of this study was to characterize the relationships between these circulating biomarkers and subclinical arterial damage, elevated blood pressure, and clinical parameters in children and adolescents with PH.

## Methods

### Patients

**Fig. 1:**
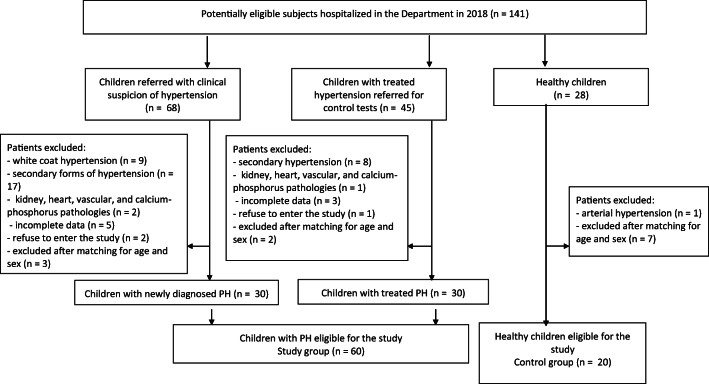
Flow diagram of the studied population (PH—primary hypertension)

This cross-sectional analysis included 60 pediatric patients with PH aged from 5.6 to 17.9 years hospitalized in one pediatric nephrology department between January and December 2018. Arterial hypertension was diagnosed according to current European guidelines [21]. The following exclusion criteria were used: secondary forms of hypertension, known kidney, heart, vascular or calcium-phosphorus pathology, as well as acute infections (temporary exclusion for 4 weeks). Twenty age- and sex-matched healthy subjects were included in the control group. The flow diagram of the participants is displayed in Fig. 1. The sample size of the study group, assessed on the basis of available literature, should be 60 (statistical power 0.80, P = 0.05).

### Calcification inhibitors and calcium-phosphorus metabolism

Venous blood was collected after 12-h fasting, centrifuged to obtain serum, and stored at − 80 °C. Concentrations of vascular calcification inhibitors were measured using enzyme-linked immunosorbent assays (ELISA) (Fetuin-A [AHSG] Human ELISA kit Ca. No. RD191037100 [μg/mL], Osteoprotegerin Human ELISA kit Cat. No. RD194003200 [pmol/L], sRANKL Human ELISA kit Cat. No. RD193004200R [pmol/L], Biovendor, Brno, Czech Republic). In addition, we assessed basic parameters of calcium-phosphorus metabolism: 25(OH)D [ng/mL], calcium [mg/dL], inorganic phosphorus [mg/dL], alkaline phosphatase [IU/L], and intact parathormone (PTH) [pg/mL]. Vitamin D concentrations were defined according to Polish recommendations [22]. Normal concentrations of calcium (8.8–10.7 mg/dL), phosphorus (2.8–5.6 mg/dL), alkaline phosphatase (45–515 IU/L), and parathormone (12–95 pg/mL) were taken from normative values according to the manufacturers’ recommendations. Vitamin D and parathormone were assessed by chemiluminescence (Alinity ci, Abbott Laboratories, Lake Bluff, IL, USA, and IMMULITE 2000XPi, Siemens Healthineers, Erlangen, Germany, respectively), all other parameters by dry chemistry (VITROS 5600, Ortho Clinical Diagnostics, Raritan, NJ, USA). Complete blood count (CBC) was performed using Coulter LH 780 hematologic analyzer (Sysmex XN1000, Sysmex Corporation, Kobe, Japan) and the following CBC-derived inflammatory indicators were evaluated: numbers of neutrophils (NEU) [1000/μL], lymphocytes (LYM; 1000/μL), platelets (PLT; 1000/μL), mean platelet volume (MPV) [fL], and neutrophil-to-lymphocyte and platelet-to-lymphocyte ratios (NLR and PLR, respectively).

### Blood pressure and subclinical arterial damage

The detailed methodology of blood pressure and arterial damage assessment was described previously [23, 24]. Peripheral office blood pressure (BP) was measured oscillometrically with Welch Allyn VSM Patient Monitor 300 (Welch Allyn Inc., Skaneateles Falls, NY, USA) [mmHg] Z-scores [25]. Twenty-four-hour ABPM was performed using the SUNTECH OSCAR 2 device (SunTech Medical, Inc., Morrisville, NC, USA). We analyzed the following ABPM-derived parameters during 24 h: systolic, diastolic, and mean arterial pressure (SBP 24 h, DBP 24 h, MAP 24 h, respectively) [mmHg], MAP 24 h Z-score, pulse pressure [mmHg], heart rate [beats per minute], SBP and DBP load [%], and nocturnal blood pressure dip [%] [26]. Central (aortic) blood pressure (AoBP) [mmHg], augmentation index normalized to heart rate of 75 beats per minute (AIx75HR) [%], subendocardial viability ratio (SEVR) [%], and aortic pulse wave velocity (PWV) [m/s] were evaluated with the Sphygmocor device (AtCor Medical Pty Ltd., Sydney, Australia) using applanation tonometry, and common carotid intima media thickness (cIMT) [mm] was measured using the Aloka Prosound Alpha 6 (Hitachi Aloka Medical, Mitaka, Japan) equipped with 13-MHz linear transducer. In addition, the following elasticity parameters of right common carotid artery (local arterial stiffness) by ECHO-tracking (ET) Aloka Prosound Alpha 6 preset were analyzed: beta (stiffness index), Ep (pressure strain elasticity modulus) [kPa], AC (arterial compliance) [mm^2^/kPa], AIx (augmentation index) [%], PWVbeta (pulse wave velocity beta) [m/s], *D*_max [mm], *D*_min [mm] (maximal and minimal diameter of the artery), DATmax (acceleration time to artery maximal diameter) [ms]. PWV, cIMT are expressed as absolute values and Z-scores using pediatric normative data [27, 28].

### Clinical and biochemical parameters

In all children, basic clinical and anthropometrical data were assessed (age [years], sex, duration of PH [months], height [cm], weight [kg], and body mass index (BMI) [kg/m^2^]); all anthropometrical parameters are presented as Z-scores [29]. Overweight and obesity were defined in accordance with World Health Organization as BMI Z-score values > 1 and > 2, respectively.

In addition, the following biochemical parameters were evaluated in all study subjects: serum creatinine [mg/dL], uric acid [mg/dL], total, low-density lipoprotein (LDL) and high-density lipoprotein (HDL) cholesterol [mg/dL], and triglycerides [mg/dL]; biochemical parameters were evaluated by dry chemistry (VITROS 5600, Ortho Clinical Diagnostics, USA). Glomerular filtration rate (GFR) was calculated according to the Schwartz formula [30].

### Statistical analysis

Statistical analysis was performed using Dell Statistica 13.0 software (TIBCO Software Inc. Palo Alto, CA, USA). Normality of data was analyzed using the Shapiro-Wilk test. The results were expressed as mean ± SD (normal distribution) or median and interquartile range (IQR) (non-normal distribution). Quantitative variables were compared for homogeneity using the Student *t* test (normal distribution) or the Mann-Whitney U test (non-normal distribution). The numbers of patients in subgroups were compared using the chi-square test and Fisher exact test. The relationship between quantitative variables were analyzed using Pearson correlation or Spearman’s rank correlation, when appropriate. Multivariate analysis was performed using forward step-wise regression analysis. Parameters that correlated with markers of arterial damage with p < 0.100 in the univariate analysis were included in the final model. Parameters that correlated with each other with r > 0.650 were excluded from the regression models so as to avoid collinearity. To detect the outliers and measure the impact on the regression equation, Cook’s distance and standardized residual values were used. The variables that caused outliers were removed from regression models. A p value of < 0.05 was considered statistically significant.

## Results

Clinical and biochemical characteristics of the studied children are shown in Table 1. PH patients did not differ from healthy children in terms of sex, age, and kidney function, as well as total and LDL cholesterol. Hypertensive children were characterized by higher BMI Z-score (1.17 (0.52–1.73) vs. 0.14 (-0.43–1.12), *P* = 0.001), uric acid (5.6 ± 1.4 vs. 4.6 ± 1.1 [mg/dL], *P* = 0.003), and triglycerides (85.5 (64–120) vs. 57.5 (49.50–71.50) [mg/dL], *P* = 0.003), and lower HDL-cholesterol (48 (42–55) vs. 58.5 (53–70) [mg/dL], *P* = 0.001) compared to the healthy ones. When we analyzed subclinical inflammation markers, we found that PH children had significantly higher NLR (1.49 (1.18–2.06) vs. 1.20 (0.89–1.53), *P* = 0.040) without differences in MPV and PLR in comparison to the control group. Among PH patients, 25 (41.7%) children were overweight, and 8 (13.3%) were obese. Duration of hypertension was 19.9 ± 25.7 (median value: 12.0, IQR: [4.0–24.5]) [months]. In the study group, half of the patients had already been treated with antihypertensive medications. Amlodipine, prescribed in 18 patients, was the most commonly used medication. Other medications were angiotensin-converting enzyme inhibitors in 11, beta-blockers in 4, angiotensin receptor blockers in 2, alpha-blockers in 1, and hydrochlorothiazide in 1 child. The vast majority of treated children received 1 antihypertensive drug, three children received 2 medications, and two children received 3 drugs. The remaining 30 children were newly diagnosed with PH and no treatment was administered yet.

**Table 1:** Clinical and biochemical characteristics of the studied population.

Analyzed parameter	Children with arterial hypertension (*n* = 60)	Healthy children (*n* = 20)	*P*
Sex (boys/girls) (n)	37/23	11/9	P = 0.609^a^
Age [years]	15.8 (14.5–16.8)	14.8 (13.0–16.4)	P = 0.207^b^
Height [cm]	170.5 (161.8–178.8)	165.0 (151.5–171.5)	P = 0.088^b^
Height Z-score	0.53 ± 1.00 (− 0.29–1.16)	0.69 ± 1.23 (0.13–1.22)	P = 0.555^c^
Weight [kg]	72.0 (61.3–84.7)	56.0 (46.0–61.0)	P < 0.001^b^
Weight Z-score	1.20 ± 0.81	0.62 ± 0.98	P = 0.010^c^
BMI [kg/m^2^]	24.7 ± 4.5	20.7 ± 3.6	P = 0.001^c^
BMI Z-score	1.17 (0.52–1.73)	0.14 (− 0.43–1.12)	P = 0.001^b^
GFR ac. to Schwartz [mL/min/1.73 m^2^]	93.9 (87.0–109.2)	101.8 (95.4–121.9)	P = 0.076^b^
Uric acid [mg/dL]	5.6 ± 1.4	4.6 ± 1.1	P = 0.003^c^
Total cholesterol [mg/dl]	150 (129–169)	148 (135–180)	P = 0.899^b^
HDL-cholesterol [mg/dL]	48 (42–55)	58.5 (53–70)	P = 0.001^b^
LDL-cholesterol [mg/dL]	83.5 (64.5–96.8)	77.3 (63.8–103.6)	P = 0.701^b^
Triglycerides [mg/dL]	85.5 (64–120)	57.5 (49.50–71.50)	P = 0.001^b^
MPV [fL]	10.6 (9.9–11.2)	11.0 (10.2–11.4)	*P* = 0.077^b^
NLR	1.49 (1.18–2.06)	1.20 (0.89–1.53)	P = 0.040^b^
PLR	130.14 ± 45.21	118.94 ± 29.28	P = 0.303^c^

*BMI* body mass index, *GFR* glomerular filtration rate, *HDL* high-density lipoprotein, *LDL* low-density lipoprotein, *MPV* mean platelet volume, *NLR* neutrophil-to-lymphocyte ratio, *PLR* platelet-to-lymphocyte ratio

^a^Fisher’s exact test

^b^Mann-Whitney *U* test

^c^Student *t* test

Peripheral blood pressure, the results of ABPM, central blood pressure, arterial stiffness parameters, cIMT, and E-tracking parameters are presented in Table 2. PH patients were characterized by significantly higher office peripheral and central blood pressures (SBP: 132.2 ± 12.2 vs. 117.0 ± 9.9 [mmHg], P < 0.001, DBP: 77.5 (73–83) vs. 67.5 (62–70) [mmHg], P < 0.001, AoSBP: 108.3 (103–117) vs. 95.5 (89–103) [mmHg], P < 0.001, AoDBP: 79.0 (74–85) vs. 69.0 (63–71) [mmHg], P < 0.001, AoMAP: 94.3 (88–99) vs. 83.5 (76–87) [mmHg], P < 0.001), ABPM blood pressure (24 h SBP: 128.4 ± 7.9 vs. 113.0 ± 6.1 [mmHg], P < 0.001, 24 h DBP: 70.0 ± 6.3 vs. 63.2 ± 3.9 [mmHg], *P* < 0.001, 24 h MAP: 89.3 ± 6.0 vs. 79.7 ± 3.5 [mmHg], P < 0.001), PWV (5.0 (4.6–5.7) vs. 4.5 (3.9–4.8) [m/s], *P* = 0.007), cIMT (0.46 ± 0.07 vs. 0.39 ± 0.03 [mm], P < 0.001), and common carotid artery maximal and minimal diameters derived from ECHO-tracking (ET D max: 6.4 ± 0.7 vs. 5.9 ± 0.7 [mm], P = 0.010, ET D min: 5.5 ± 0.7 vs. 5.1 ± 0.7 [mm], P = 0.021). At the moment of evaluation, elevated (i.e., equal or above 95^th^ percentile) office systolic blood pressure was found in 31 (51.7%) and diastolic in 50 (83.3%) children with PH; elevated ABPM systolic blood pressure was revealed in 42 (83.3%), diastolic in 9 (15.0%), and MAP in 13 (21.7%) patients with PH. Also, in the group of patients with PH, abnormal PWV was found in 5 (8.3%) and cIMT in 29 (48.3%) patients.

**Table 2:** Blood pressure and parameters of arterial damage in children with primary hypertension and in control group

Analyzed parameter	Children with arterial hypertension (n = 60)	Healthy children (*n* = 20)	P
SBP [mmHg]	132.2 ± 12.2	117.0 ± 9.9	*P* < 0.001^a^
SBP Z-score	2.30 ± 0.94	0.65 ± 0.86	*P* < 0.001^a^
DBP [mmHg]	77.5 (73–83)	67.5 (62–70)	*P* < 0.001^b^
DBP Z-score	1.51 ± 0.91	0.18 ± 0.67	*P* < 0.001^a^
AoSBP [mmHg]	108.3 (103–117)	95.5 (89–103)	*P* < 0.001^b^
AoDBP [mmHg]	79.0 (74–85)	69.0 (63–71)	*P* < 0.001^b^
AoMAP [mmHg]	94.3 (88–99)	83.5 (76–87)	*P* < 0.001^b^
24h SBP [mmHg]	128.4 ± 7.9	113.0 ± 6.1	*P* < 0.001^a^
24h DBP [mmHg]	70.0 ± 6.3	63.2 ± 3.9	*P* < 0.001^a^
24h MAP [mmHg]	89.3 ± 6.0	79.7 ± 3.5	*P* < 0.001^a^
24h MAP Z-score	0.90 (0.17–1.61)	-0.54 (-0.87–0.08)	*P* < 0.001^b^
24h SBPL [%]	37.5 (22–55)	6.9 (4–16)	*P* < 0.001^b^
24h DBPL [%]	17.5 (8–26)	7.0 (1–10)	*P* < 0.001^b^
SBP DIP [%]	10.8 ± 5.0	10.3 ± 4.0	*P* = 0.657^a^
DBP DIP [%]	15.9 ± 7.5	15.7 ± 5.5	*P* = 0.943^a^
AIx75HR [%]	− 6.3 (− 11.5–4.8)	− 3.0 (− 9.2–2.2)	*P* = 0.685^b^
SEVR [%]	149.8 (133–186)	153.5 (136–172)	*P* = 0.859^b^
PWV [m/s]	5.0 (4.6–5.7)	4.5 (3.9–4.8)	*P* = 0.007^b^
PWV Z-score	− 0.18 ± 1.18	− 0.99 ± 0.94	*P* = 0.007^a^
cIMT [mm]	0.46 ± 0.07	0.39 ± 0.03	*P* < 0.001^a^
cIMT Z-score	1.44 ± 1.40	0.16 ± 0.57	*P* < 0.001^a^
ET beta	3.5 (2.7–4.3)	3.6 (3.2–4.6)	*P* = 0.487^b^
ET Ep [kPa]	46.5 (35–59)	42.0 (35–53)	*P* = 0.512^b^
ET AC [mm^2^/kPa]	1.1 (0.9–1.5)	1.0 (0.8–1.2)	*P* = 0.220^b^
ET AIx [%]	− 2.2 (− 8.7–1.3)	− 3.2 (− 8.0 to − 1.3)	*P* = 0.512^b^
ET PWVbeta [m/s]	4.0 (3.5–4.7)	3.9 (3.5–4.3)	*P* = 0.453^b^
ET D max [mm]	6.4 ± 0.7	5.9 ± 0.7	*P* = 0.010^a^
ET D min [mm]	5.5 ± 0.7	5.1 ± 0.7	*P* = 0.021^a^
ET DATmax [ms]	127.0 (108–146)	130.5 (126–150)	*P* = 0.167^b^

*SBP* systolic blood pressure, *DBP* diastolic blood pressure, *AoSBP* aortic (central) systolic blood pressure, AoDBP aortic (central) diastolic blood pressure, *AoMAP* aortic (central) mean blood pressure, *SBPL* systolic blood pressure load, *DBPL* diastolic blood pressure load, *AIx75HR* augmentation index normalized to heart rate of 75 beats per minute, *SEVR* subendocardial viability ratio, *PWV* pulse wave velocity, *cIMT* common carotid artery intima-media thickness, *ET* ECHO-tracking, *beta* stiffness index, *Ep* pressure strain elasticity modulus, *AC* arterial compliance, *AIx* augmentation index, *D*_*max* maximal diameter of the artery, *D*_*min* minimal diameter of the artery, *DATmax* acceleration time to artery maximal diameter

^a^Student *t* test

^b^Mann-Whitney *U* test

**Fig. 2:**
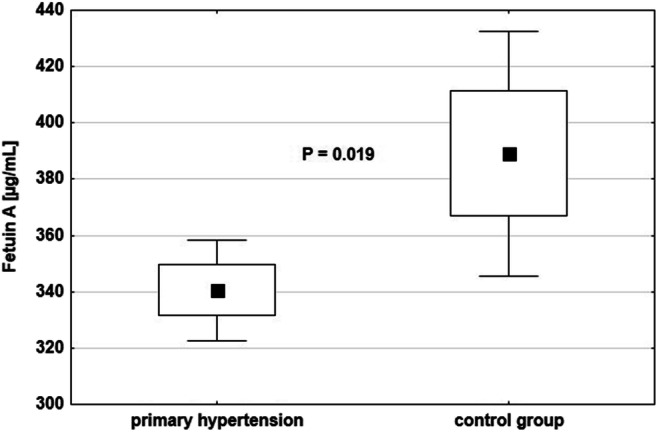
Serum concentrations of fetuin A in children with primary hypertension and in control group

Concentrations of circulating calcification inhibitors and parameters of calcium and phosphorus metabolism are presented in Table 3. PH patients were characterized by significantly lower FA level (340.57 ± 70.43 vs. 389.19 ± 99.22 [μg/mL], P = 0.019) (Fig. 2) without differences in OPG, sRANKL, and OPG/sRANKL and OPG/FA ratios. Calcium and phosphorus metabolism parameters remained within normal limits in all the studied children except for vitamin D concentrations. Vitamin D high supply (25(OH)D > 50–100 ng/mL) was found in 1 (1.7%) child with PH, vitamin D optimal status (25(OH)D > 30–50 ng/mL) in 2 (3.3%) patients with PH and in 2 (10.0%) healthy children, vitamin D suboptimal status (25(OH)D 20–30 ng/mL) in 22 (36.7%) patients with PH and in 8 (40.0%) healthy individuals, vitamin D deficiency (25(OH)D < 20–> 10 ng/mL) in 32 (53.3%) patients with PH and in 10 (50.0%) healthy children; severe vitamin D deficiency (25(OH)D < 10 ng/mL) was revealed in 3 (5.0%) hypertensive children. Despite normocalcemia in all the studied children, serum calcium concentration was significantly higher in patients with PH compared to the control group (10.0 ± 0.3 vs. 9.8 ± 0.3 [mg/dL], *P* = 0.011).

**Table 3:** Markers of bone turnover and calcium-phosphorus metabolism parameters in children with primary hypertension and in healthy children

Analyzed parameter	Children with arterial hypertension (*n* = 60)	Healthy children (*n* = 20)	*P*
Fetuin A [μg/mL]	340.57 ± 70.43	389.19 ± 99.22	*P* = 0.019^a^
OPG [pmol/L]	3.89 ± 0.86	4.08 ± 0.86	*P* = 0.412^a^
sRANKL [pmol/L]	113.41 (89.74–173.25)	139.00 (92.17–224.11)	*P* = 0.474^b^
OPG/sRANKL * 100 [pmol/L]/[pmol/L]	3.43 (1.94–4.83)	2.58 (2.00–4.31)	*P* = 0.621^b^
OPG/Fetuin A * 100 [pmol/L]/[μg/mL]	1.19 ± 0.36	1.11 ± 0.34	*P* = 0.359^a^
Calcium [mg/dL]	10.0 ± 0.3	9.8 ± 0.3	*P* = 0.011^a^
Phosphorus [mg/dL]	4.4 ± 0.7	4.5 ± 0.4	*P* = 0.383^a^
Calcium phosphorus product [mg^2^/dL^2^]	43.5 ± 7.2	43.9 ± 3.9	*P* = 0.840^a^
Parathormone [pg/mL]	24.3 (16.9–35.5)	19.7 (14.9–31.2)	*P* = 0.230^b^
Alkaline phosphatase [IU/L]	107.0 (80–153)	152.0 (93–197)	*P* = 0.115^b^
25(OH)D [ng/mL]	19.3 (15.6–22.7)	20.6 (15.8–25.9)	*P* = 0.527^b^

*OPG* osteoprotegerin, *sRANKL* soluble ligand of the receptor activator of nuclear factor kappa-B

^a^Student *t* test

^b^Mann-Whitney *U* test

In the group of 60 children with PH, the levels of FA, OPG, sRANKL, and OPG/sRANKL and OPG/FA ratios did not differ significantly between boys and girls, and between those treated and not treated with antihypertensive medications.

**Fig. 3:**
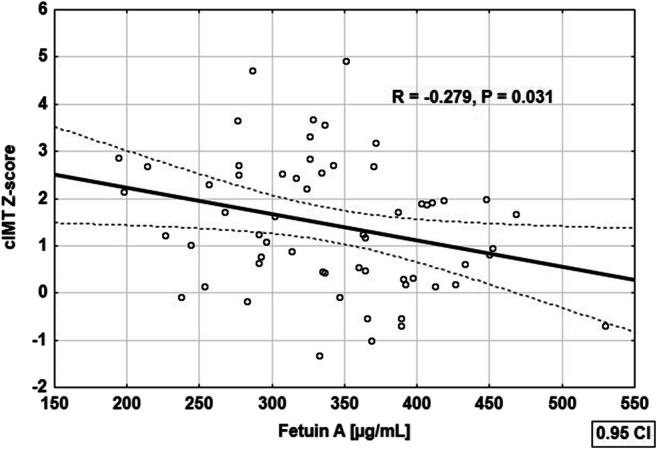
Correlation between serum fetuin A concentration and common carotid artery intima media thickness in children with primary hypertension

The correlations of the analyzed biomarkers with clinical and biochemical parameters in children with PH are presented in Table 4. In hypertensive children, FA level correlated positively with serum calcium concentration (*R* = 0.389, *P* = 0.002), systolic blood pressure nighttime dipping (*R* = 0.266, *P* = 0.040), and negatively with cIMT (*R* = − 0.279, *P* = 0.031) (Fig. 3), ECHO-tracking augmentation index (*R* = − 0.453, *P* < 0.001), and DAT max (*R* = − 0.373, *P* = 0.003). OPG level correlated positively with NLR (*R* = 0.264, *P* = 0.041) and PLR (*R* = 0.257, *P* = 0.047), and negatively with ECHO-tracking *D*_max (*R* = − 0.263, *P* = 0.043). sRANKL level correlated negatively with systolic blood pressure (*R* = − 0.270, *P* = 0.037), systolic and diastolic blood pressure loads in ABPM (*R* = − 0.390, *P* = 0.002 and *R* = − 0.266, *P* = 0.040, respectively), and augmentation index (*R* = − 0.291, *P* = 0.024). OPG/sRANKL ratio correlated positively with systolic blood pressure load in ABPM (*R* = 0.350, *P* = 0.006), and OPG/FA ratio with local arterial stiffness (ET) parameters: augmentation index (*R* = 0.297, *P* = 0.021) and *D*_max (*R* = − 0.268, *P* = 0.039). No additional correlations were found in treated and untreated patients.

**Table 4:** Correlations of biomarkers of bone metabolism with clinical and biochemical parameters in children with primary hypertension. Significant correlations (*P* < 0.05) are shown in bold

	FA	OPG	sRANKL	OPG/sRANKL	OPG/FA
NLR	*R* = 0.005^a^	***R*** = **0.264**^a^	*R* = − 0.074^a^	*R* = 0.171^a^	*R* = 0.182^a^
	*P* = 0.704	*P* = **0.041**	*P* = 0.572	*P* = 0.191	*P* = 0.164
PLR	*R* = − 0.025^b^	***R*** = **0.257**^b^	*R* =0.064^a^	*R* = 0.043^a^	*R* = 0.213^b^
	*P* = 0.851	*P* = **0.047**	*P* = 0.626	*P* = 0.747	*P* = 0.102
Calcium [mg/dL]	**R** = **0.389**^b^	*R* = 0.051^2^	*R* = 0.082^a^	*R* = − 0.054^a^	*R* = − 0.251^b^
	*P* = **0.002**	*P* = 0.697	*P* = 0.533	*P* = 0.683	*P* = 0.053
ABPM SBP 24 h [mmHg]	*R* = − 0.106^b^	*R* = − 0.007^b^	***R*** = − **0.270**^a^	*R* = 0.187^a^	*R* = 0.008^b^
	*P* = 0.420	*P* = 0.960	*P* = **0.037**	*P* = 0.152	*P* = 0.951
ABPM SBPL 24 h [%]	*R* = 0.025^a^	*R* = 0.067^a^	***R*** = − **0.390**^a^	***R*** = **0.350**^a^	*R* = 0.090^a^
	*P* = 0.850	*P* = 0.613	*P* = **0.002**	*P* = **0.006**	*P* = 0.492
ABPM DBPL 24 h [%]	*R* = − 0.001^a^	*R* = − 0.018^a^	***R*** = − **0.266**^a^	*R* = 0.200^a^	*R* = 0.012^a^
	*P* = 0.993	*P* = 0.889	*P* = **0.040**	*P* = 0.126	*P* = 0.925
ABPM SBP DIP	***R*** = **0.266**^b^	*R* = 0.016^b^	*R* = − 0.061^a^	*R* = 0.072^a^	*R* = − 0.152^b^
	*P* = **0.040**	*P* = 0.901	*P* = 0.645	*P* = 0.584	*P* = 0.246
AIx75HR [%]	*R* = 0.087^a^	*R* = − 0.102^a^	***R*** = − **0.291**^a^	*R* = 0.208^a^	*R* = − 0.130^a^
	*P* = 0.510	*P* = 0.437	*P* = **0.024**	*P* = 0.110	*P* = 0.322
cIMT [mm]	***R*** = − **0.279**^b^	*R* = 0.007^b^	*R* = − 0.025^a^	*R* = 0.043^a^	*R* = 0.176^b^
	*P* = **0.031**	*P* = 0.958	*P* = 0.851	*P* = 0.746	*P* = 0.179
cIMT Z-score	***R*** = − **0.281**^b^	*R* = 0.039^b^	*R* = − 0.021^a^	*R* = 0.044^a^	*R* = 0.194^b^
	*P* = **0.030**	*P* = 0.770	*P* = 0.874	*P* = 0.738	*P* = 0.137
ET AIx [%]	***R*** = − **0.453**^a^	*R* = − 0.069^a^	*R* = − 0.017^a^	*R* = − 0.009^a^	***R*** = **0.297**^a^
	*P* < **0.001**	*P* = 0.598	*P* = 0.895	*P* = 0.948	*P* = **0.021**
ET D_max [mm]	*R* = 0.130^b^	***R*** = − **0.263**^b^	*R* = − 0.088^a^	*R* = − 0.008^a^	***R*** = − **0.268**^b^
	*P* = 0.321	*P* = **0.043**	*P* = 0.501	*P* = 0.949	*P* = **0.039**
ET DAT max [ms]	***R*** = − **0.373**^a^	*R* = − 0.114^a^	*R* = 0.146^a^	*R* = − 0.176^a^	*R* = 0.177^a^
	*P* = **0.003**	*P* = 0.385	*P* = 0.266	*P* = 0.179	*P* = 0.177

*FA* fetuin A, *OPG* osteoprotegerin, *sRANKL* soluble ligand of the receptor activator of nuclear factor kappa-B, *NLR* neutrophil-to-lymphocyte ratio, *PLR* platelet-to-lymphocyte ratio, *ABPM* ambulatory blood pressure monitoring, *SBPL* systolic blood pressure load, *DBPL* diastolic blood pressure load, *AIx75HR* augmentation index normalized to heart rate of 75 beats per minute, *cIMT* common carotid artery intima-media thickness, *ET* ECHO-tracking, *AIx* augmentation index, *D*_*max* maximal diameter of the artery, *DATmax* acceleration time to artery maximal diameter

^a^Spearman’s rank correlation

^b^Pearson correlation

In healthy children, positive correlations were observed between sRANKL, OPG/sRANKL ratio, and body measurement parameters (sRANKL vs. height Z-score: *R* = 0.540, *P* = 0.014; sRANKL vs. weight Z-score: *R* = 0.549, *P* = 0.012, sRANKL vs. BMI Z-score: *R* = 0.481, *P* = 0.032, OPG/sRANKL vs. height Z-score: *R* = − 0.606, *P* = 0.005, OPG/sRANKL vs. weight Z-score: *R* = − 0.477, *P* = 0.034), between OPG and total cholesterol and (*R* = 0.452, *P* = 0.045) and between OPG/FA ratio and total and HDL-cholesterol (*R* = 0.546, *P* = 0.013 and *R* = 0.578, *P* = 0.008, respectively). In addition, OPG and sRANKL correlated positively with 25OHD level (*R* = 0.463, *P* = 0.040 and *R* = 0.540, *P* = 0.014, respectively); FA and FA/OPG ratio correlated positively with PTH (*R* = 0.616, *P* = 0.004 and *R* = − 0.672, *P* = 0.001, respectively). Correlations were observed also between FA, OPG/FA ratio, and AIx75HR (*R* = − 0.589, *P* = 0.006, *R* = 0.564, *P* = 0.010, respectively), and between OPG/FA, AoPP, and SEVR (*R* = − 0.477, *P* = 0.033, *R* = − 0.465, *P* = 0.039). A table with correlations in the control group is included in the Supplementary Materials.

In multivariate analysis using forward step-wise regression, we found that in the entire group of 80 children, FA was a significant determinant for both cIMT [mm] and cIMT Z-score (*R*^2^ = 0.270 and *R*^2^ = 0.192, respectively) (Table 5).

**Table 5:** Results of multivariate analysis by forward step-wise regression of determinants of markers of arterial damage in children with primary hypertension

	Beta	95%CI	*P*
cIMT [mmHg]			
Fetuin A [μg/mL]	− 0.208	(− 0.407 to − 0.009)	*P* = 0.041
SBP [mmHg]	0.347	(0.146 to 0.548)	*P* < 0.001
cIMT Z-score			
Fetuin A [μg/mL]	− 0.224	(− 0.435 to − 0.013)	*P* = 0.038
SBP Z-score	0.268	(0.054 to 0.482)	*P* = 0.015

*CI* confidence interval, *cIMT* common carotid artery intima-media thickness, *SBP* systolic blood pressure

## Discussion

Our single center cross-sectional analysis revealed that children with primary hypertension were characterized by lower fetuin A (FA) serum concentration, without differences in OPG, soluble ligand of the receptor activator of nuclear factor kappa-B (sRANKL), and OPG/FA and OPG/sRANKL ratios. We showed that low FA and sRANKL levels and high OPG/FA and OPG/sRANKL ratios were related to elevated blood pressure and subclinical arterial damage in patients with primary hypertension. In addition, high OPG/FA ratio may be associated with arterial stiffness also in healthy individuals.

A large proportion of hypertensive patients were characterized by the presence of subclinical arterial damage (early sign of hypertensive arteriosclerosis)—increased cIMT, despite a relatively short duration of hypertension (mean 1.5 years). We cannot exclude the possibility that in many of our patients the disease lasted longer but the diagnosis was delayed due to a silent clinical course or nonspecific symptoms. Similar to our results, other authors also found that subclinical arterial damage is not rare in this group of patients [4, 31]. These findings highlight the fact that PH is by far not a benign phenomenon and carries a significant risk for cardiovascular sequelae in adult life. Our PWV results have been compared with normative values obtained from other devices also using the principle of applanation tonometry [27]. Of note, Stabouli et al. [32] and Kis et al. [33] found that the most frequently used techniques of measurement of aortic PWV provide comparable results and can be used interchangeably in the pediatric setting.

As high blood pressure influences various parts of the arterial tree to different extents, the assessment of local stiffness may provide additional information on target organ damage in hypertensive individuals. The assessment of carotid stiffness is of particular interest as stiffening of the carotid arteries increases pressure and flow pulsatility in the cerebral circulation and puts the patient at risk of a stroke and its sequelae. The assessment of carotid stiffness may be performed either by routine ultrasound examination in M-mode [28] or by high-resolution wall tracking systems analyzing the raw radiofrequency, e.g., E-tracking of Hitachi-Aloka [34]. Mean values of indices of local stiffness (beta, Ep, AIx, PWVbeta) were higher in children with PH but the difference did not reach statistical significance. Increased carotid stiffness had already been found in children with overweight and obesity (measured by E-tracking) [35] and with hypertension [4]. It is noteworthy that both these studies showed increased systolic and diastolic carotid artery dimensions in patients with cardiovascular risk factors, similar to our PH cohort [4, 35]. Carotid arterial diameter enlargement is a manifestation of arterial wall remodeling and is considered a risk factor for stroke, cardiovascular disease, and mortality [36].

Fetuin A, a member of the cystatin superfamily of cysteine protease inhibitors, is a negative acute-phase glycoprotein produced by the liver. It is a potent inhibitor of extraosseous calcification and is regarded as a marker for vascular inflammation [6, 10]. Our hypertensive patients were characterized by significantly lower fetuin A serum concentrations compared to their healthy peers. Similarly, in patients with PH, FA level correlated negatively with cIMT and arterial stiffness evaluated by E-tracking. In healthy controls, FA correlated negatively with AIx75HR but not with cIMT. Interestingly, in FA-knock-out mice, the aorta was unaltered, whereas the peripheral vessels in the skin and kidney were affected by extensive calcification [10]. Though the direct role of fetuin A in the process of arteriosclerosis is not known, it is hypothesized that FA Ca-P complex (calcyprotein) interacts with cell membrane proteins — annexin II and VI — and inhibits transformation of vascular smooth vascular cells (VSMC) into osteoblast-like cells, and inhibits the function of bone morphogenetic protein 2 [6, 10, 12]. In addition, FA and type II TGFβ-receptors have a sequence homology at the cytokine binding site. Thus, FA interacts with TGFbeta signaling and influences collagen and extracellular matrix production and arterial stiffness [37].

Numerous studies show that low fetuin A is related to increased risk of arterial damage and cardiovascular disease. Our results are consistent with the study by Guarneri et al. who also found lower FA level in adults with PH (mean age 46.9 ± 11.8 years) compared to their healthy peers, and showed negative correlation between FA and cIMT [15]. Negative correlations of fetuin A with cIMT [16] and arterial stiffness (evaluated as PWV and AIx75HR) [17, 18, 38] were also found in children with CKD. Finally, low plasma FA levels were associated with an increased risk of all-cause and CVD mortality in patients with coronary artery disease in a Chinese population [13]. Nevertheless, the final position of FA as a negative biomarker of cardiovascular burden is not yet confirmed, as numerous studies have shown no significant relationship between FA and cardiovascular injury markers in various groups of high-risk pediatric patients, e.g., in obese adolescents [19] or in children after kidney transplantation [20].

In our group, fetuin A level was positively correlated with serum calcium, but only in hypertensive patients. Similar dependence was revealed by Makulska et al. in children with CKD [17]. It is possible that in patients with increased cardiovascular risk, the level of FA increases alongside calcium concentration as a preventive measure against soft tissue calcification. FA level negatively correlates with subclinical inflammation markers and is considered a negative inflammatory reactant. In adult patients with PH, FA level correlated negatively with inflammatory cytokines: tumor necrosis factor alpha and interleukin 6 [15]. PH is nowadays considered as a state of subclinical inflammation [1]. In line with this hypothesis, our hypertensive patients were characterized by a higher level of CBC-derived markers of subclinical inflammation — NLR — as compared to their healthy peers. The down-regulation of FA in inflammatory states, such as in the course of PH, might be the mechanism facilitating early vascular damage in these individuals.

In vitro and animal studies suggest that OPG acts as a calcification inhibitor; however, the direct mechanism underlying its inhibitory action is not yet described. The vessel wall is a site of abundant OPG production, and in line with this OPG knock-out mice exhibit aorta and renal artery calcification [11]. In our cohort of hypertensive patients, OPG level was not directly related to blood pressure or arterial damage, but sRANKL level showed a negative relationship with blood pressure and arterial stiffness.

The vascular role of OPG is probably multifaceted and includes interaction with its ligands, with TNFα, and with NF-kappaB pathway. In addition, OPG inhibits alkaline phosphatase–mediated osteogenic differentiation of vascular cells [8, 9]. However, clinical studies suggest that serum OPG increases along with the extent of vascular calcification. It is hypothesized that excessive vascular lesions induce further production of OPG to compensate for the process of vascular calcification. OPG level was significantly elevated in adults with PH and increased arterial stiffness [14, 39], and is considered an independent risk factor of cardiovascular risk in adults [40]. However, data on circulating sRANKL concentrations are sparse and contradictory. A positive relationship between sRANKL and the risk of cardiovascular events was revealed, but not with cIMT, suggesting different pathway to vascular damage [41]. The authors of the aforementioned study hypothesized that either sRANKL was related to unstable plaques and not to arteriosclerotic burden in general, or the increased sRANKL level was a consequence of plaque inflammation [36, 41]. Similarly, Shroff et al. found a positive correlation between OPG and PWV without any correlation between OPG/sRANKL, sRANKL, and arterial damage parameters in children with CKD [38]. Also, Greek authors found no relationship between OPG, sRANKL, and cIMT in children and adolescents with type 1 diabetes mellitus [42]. Contrary to those studies, our results suggest that high sRANKL levels could exert a protective role against blood pressure elevation and arterial stiffness in hypertensive patients. Further, prospective studies are needed to confirm this in other groups of patients and to unmask the underlying mechanism.

Of note, in patients with PH, we have demonstrated a positive correlation between OPG and CBC-derived markers of subclinical inflammation — NLR and PLR. OPG is steadily released from vascular endothelial cells in response to inflammatory stimuli, suggesting that it plays a modulatory role in vascular injury, inflammation, and atherosclerosis [43]. OPG level positively correlates with CRP in adult patients with metabolic syndrome [44], and TNFα together with IL-1β stimulated production of OPG by human retinal microvascular endothelial cells [45]. Our results suggest that subclinical inflammation might be the link between OPG/RANK/sRANKL axis, elevation of blood pressure, and target organ damage in pediatric patients with PH.

The particular strength of our study lies in the extensive evaluation of blood pressure and numerous markers of arterial damage evaluated by different techniques, as well as detailed biochemical evaluation performed in both hypertensive and normotensive children. These detailed characteristics allow us to get better insight into the role of three molecules, namely FA, OPG, and sRANKL, associated with vascular calcification. The main limitations are the small number of analyzed children (especially in the control group, i.e., 20 children) and the cross-sectional design of the study, which prevents obtaining solid conclusions on causal relationships between FA, OPG, sRANKL levels, and the analyzed parameters. Additionally, subclinical inflammation was evaluated only on the basis of CBC-derived parameters with indefinite clinical significance, and we have not evaluated more precise markers like high-sensitivity C-reactive protein (hsCRP). Also, some literature data suggest that calcification inhibitors might be related to body composition — we found only a positive correlation between sRANKL and BMI Z-score in healthy children, but we have not performed deeper anthropological analysis (e.g., waist and hip measurements). Despite these limitations, our findings can be informative for larger population studies of cardiovascular disease in young patients with PH. Further prospective and interventional studies are needed to evaluate possible preventive measures against calcification-mediated early arterial damage in these patients.

## Conclusions

This study shows for the first time that in children with PH arterial damage is related to lower fetuin A concentrations.

## Supplementary Information

ESM 1 (PPTX 66 kb)

ESM 2 (XLSX 65 kb)

ESM 3 (DOCX 16 kb)

## Data Availability

All data generated or analyzed during this study are included in this published article and its supplementary information files.
