# Supplementary material for: Circulating calcification inhibitors are associated with arterial damage in pediatric patients with primary hypertension
Source: Pediatr Nephrol. 2021 Feb 18;36(8):2371–82. doi: 10.1007/s00467-021-04957-5 (PMC8260424; doi:10.1007/s00467-021-04957-5)
Supplement: Supplementary file 1 — (PPTX 66 kb) [file 467_2021_4957_MOESM1_ESM.pptx]

## Slide 1
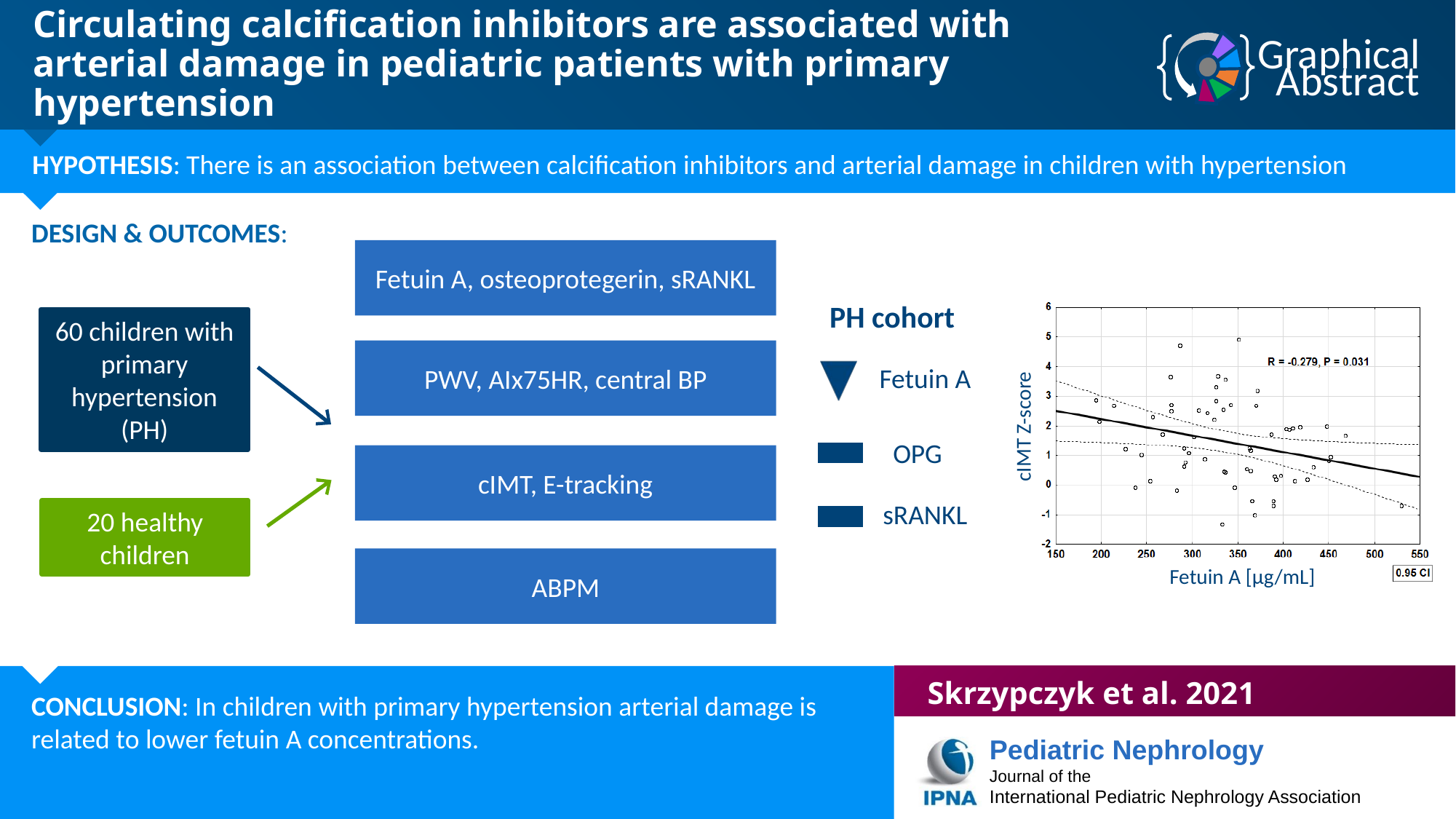

Circulating calcification inhibitors are associated with arterial damage in pediatric patients with primary hypertension
HYPOTHESIS: There is an association between calcification inhibitors and arterial damage in children with hypertension
DESIGN & OUTCOMES:
Fetuin A, osteoprotegerin, sRANKL
cIMT Z-score
Fetuin A [μg/mL]
PH cohort
60 children with primary hypertension
(PH)
PWV, AIx75HR, central BP
Fetuin A
OPG
cIMT, E-tracking
sRANKL
20 healthy children
ABPM
Skrzypczyk et al. 2021
CONCLUSION: In children with primary hypertension arterial damage is related to lower fetuin A concentrations.
