# Supplementary material for: Circulating calcification inhibitors are associated with arterial damage in pediatric patients with primary hypertension
Source: Pediatr Nephrol. 2021 Feb 18;36(8):2371–82. doi: 10.1007/s00467-021-04957-5 (PMC8260424; doi:10.1007/s00467-021-04957-5)
Supplement: Supplementary file 3 — (DOCX 16 kb) [file 467_2021_4957_MOESM3_ESM.docx]

Table 5

Correlations of biomarkers of bone metabolism with clinical and biochemical parameters in healthy children. Significant correlations (p>0.05) are shown in bold.

|  | **FA** | **OPG** | **sRANKL** | **OPG/sRANKL** | **OPG/FA** |
| --- | --- | --- | --- | --- | --- |
| **Height Z-score** | R=0.172^1^  P=0.468 | R=0.020^1^  P=0.934 | **R=0.540**^2^  **P=0.014** | **R=-0.606**^2^  **P=0.005** | R=-0.127^1^  P=0.593 |
| **Weight Z-score** | R=-0.059^1^  P=0.806 | R=0.147^1^  P=0.535 | **R=0.549**^2^  **P=0.012** | **R=-0.477**^2^  **P=0.034** | R=0.098^1^  P=0.682 |
| **BMI Z-score** | R=-0.187^1^  P=0.431 | R=0.300^1^  P=0.198 | **R=0.481**^2^  **P=0.032** | R=-0.361^2^  P=0.118 | R=0.280^1^  P=0.232 |
| **PLR** | **R=-0.463**^1^  **P=0.040** | R=-0.196^1^  P=0.407 | R=-0.271^2^  P=0.248 | R=0.186^2^  P=0.431 | R=0.152^1^  P=0.523 |
| **Total cholesterol**  **[mg/dL]** | R=-0.328^1^  P=0.158 | **R=0.452**^1^  **P=0.045** | R=0.394^2^  P=0.085 | R=-0.307^2^  P=0.188 | **R=0.546**^1^  **P=0.013** |
| **HDL cholesterol [mg/dL]** | R=-0.351^1^  P=0.129 | R=0.401^1^  P=0.080 | R=0.132^2^  P=0.578 | R=-0.099^2^  P=0.679 | **R=0.578**^1^  **P=0.008** |
| **25OHD [ng/mL]** | R=-0.065^2^  P=0.784 | **R=0.463**^2^  **P=0.040** | **R=0.540**^2^  **P=0.014** | R=-0.467^2^  P=0.038 | R=0.347^2^  P=0.134 |
| **parathormone [pg/mL]** | **R=0.616**^2^  **P=0.004** | R=-0.239^2^  P=0.311 | R=-0.429^2^  P=0.059 | R=0.402^2^  P=0.079 | **R=-0.672**^2^  **P=0.001** |
| **AoPP [mm Hg]** | R=0.312^1^  P=0.181 | R=-0.226^1^  P=0.338 | R=-0.039^2^  P=0.871 | R=0.074^2^  P=0.757 | **R=-0.477**^1^  **P=0.033** |
| **AIx75HR [%]** | **R=-0.589**^2^  **P=0.006** | R=0.136^2^  P=0.569 | R=0.263^2^  P=0.262 | R=-0.203^2^  P=0.390 | **R=0.564**^2^  **P=0.010** |
| **SEVR [%]** | R=0.341^1^  P=0.142 | R=-0.246^1^  P=0.296 | R=-0.035^2^  P=0.885 | R=-0.005^2^  P=0.985 | **R=-0.465**^1^  **P=0.039** |
| **ET beta** | **R=-0.475**^1^  **P=0.034** | R=0.026^1^  P=0.912 | R=-0.049^2^  P=0.837 | R=0.118^2^  P=0.619 | R=0.387^1^  P=0.092 |

FA – fetuin A, OPG – osteoprotegerin, sRANKL - soluble ligand of the receptor activator of nuclear factor kappa-B, BMI – body mass index, PLR – platelet-to-lymphocyte ratio, HDL – high density lipoprotein, AoPP – aortic (central) pulse pressure, AIx75HR – augmentation index normalized to heart rate of 75 beats per minute, SEVR – subendocardial viability ratio, ET – ECHO-tracking, beta - stiffness index

^1^ - Pearson correlation

^2^ - Spearman’s rank correlation
